# Association of serum lipid levels and prostate cancer severity among Hispanic Puerto Rican men

**DOI:** 10.1186/s12944-015-0096-0

**Published:** 2015-09-17

**Authors:** Jeannette Salgado-Montilla, Marievelisse Soto Salgado, Barbara Surillo Trautmann, Ricardo Sánchez-Ortiz, Margarita Irizarry-Ramírez

**Affiliations:** UPR/MDACC Partnership in Excellence in Cancer Research Program, Medical Sciences Campus, University of Puerto Rico, PO Box 365067, San Juan, 00936-5067 Puerto Rico; Graduate Department, Clinical Laboratory Sciences, School of Health Professions, Medical Sciences Campus, University of Puerto Rico, PO Box 365067, San Juan, 00936-5067 Puerto Rico; Urology Section, School of Medicine, Medical Sciences Campus, University of Puerto Rico, PO Box 365067, San Juan, 00936-5067 Puerto Rico

**Keywords:** Triglycerides, Prostate cancer, Cholesterol, Obesity, Hispanics

## Abstract

**Background:**

While obesity and fat intake have been associated with an increased risk of prostate cancer (PCa) aggressiveness and mortality, the association between lipid levels and PCa phenotype remains unclear. Previous reports evaluating this association are inconsistent and highly variable when considering different racial/ethnic groups. There are scarce data regarding this association among Hispanics, and specifically Puerto Rico’s Hispanic men, a population with a higher burden of PCa, metabolic syndrome and overweight. This population has a different ancestry profile than other Hispanics from Central and South America. Due to the above the researchers inquired if there is a relationship between serum lipid levels and PCa phenotype in this understudied population using a cohort of patients treated with radical prostatectomy as their first treatment.

**Methods:**

We performed an exploratory retrospective medical record review study of 199 PCa patients who underwent radical prostatectomy between 2005 and 2012. Variables analyzed included age at PCa diagnosis, Body Mass Index (BMI), preoperative serum prostate-specific antigen (PSA), lipid levels, and clinical parameters such as prostatectomy pathologic stage and Gleason Score (GS). PCa severity was defined using pathologic stage and GS. Unadjusted and adjusted logistic regression models were fitted to estimate the odds ratios (ORs) with 95 % confidence intervals (CI) to define the relationship among clinical characteristics and PCa severity.

**Results:**

Mean age for the cohort was 58.8 years (range: 40–75), 78.9 % were overweight or obese, 36.7 % had hypertriglyceridemia, and 35.2 % had low HDL levels. In the unadjusted logistic regression model, hypertriglyceridemia (OR: 2.11, 95 % CI = 1.13–3.93), low HDL (OR: 1.90, 95 % CI = 1.02–3.56-), and age (OR: 2.34, 95 % CI 1.25–4.40) were significantly associated with a diagnosis of high severity of PCa.

**Conclusions:**

In Puerto Rican men with PCa, elevated hypertriglyceridemia, low HDL levels, and age were statistically associated with high grade PCa on bivariate analysis. Total cholesterol level was not associated with severity of disease. Associations lost significance upon multivariate adjustment. These data generate important hypotheses regarding the potential relationship between lipid pathways and PCa development and underscore the need to perform larger scale and longitudinal studies to sort out whether, hypertriglyceridemia is associated with PCa phenotype and development.

## Background

In Puerto Rico, the age-adjusted incidence rate of prostate cancer (PCa) between 2008 and 2012 was 152.8 per 100,000 men, while the age-adjusted death rate for the same period was 30.6 per 100,000 men [1]. Of the 78 municipalities in the Island, 30 had an age-adjusted death rate of 35 or higher, which highlights a possible health disparity. Also for this period, prostate cancer was the leading cause of death by cancer in men accounting for 17.6 % of all cancer deaths in men. These statistics show that PCa mortality in Puerto Rican men is higher than in Caucasians and continental United States (US) Hispanics [2–5], and warrants a study to identify the underlying causes of this high death rate.

Previous data have shown that risk factors associated with the development of PCa include age and family history. In addition a high dietary intake of animal fat or meat, and obesity seems to be related to an increase in the risk of developing the disease or its aggressiveness [6, 7]. While studies evaluating the association between obesity and PCa have been conflicting, most suggest that obesity has a positive correlation with a higher risk of developing high-grade PCa [8] and dying of PCa [9]. In obese men, abdominal adiposity may be related to progression of existing disease and biochemical recurrence after treatment [10].

In Puerto Rico, according to statistics of the Behavioral Risk Factor Surveillance System (BRFSS) for 2012, 37.8 % of citizens were overweight and 28.4 % were obese [11]. The BRFSS also indicates that 39 % of the population had high cholesterol levels. Moreover, a cross-sectional study evaluating the prevalence of metabolic syndrome in the metropolitan area of San Juan Puerto Rico, established that the prevalence of metabolic syndrome correlated with increasing age, rising to 58.2 % in participants between the ages of 70 and 80. Moreover, the researchers found that in their study cohort, men had higher levels in triglycerides than women [12].

Previous work by our group in 2010, established that Puerto Rican Hispanic men with a higher BMI had a higher prevalence of metastatic disease than those with a low BMI. In addition, we also reported that obese or overweight men with low levels of PSA had positive prostate biopsies, making this a population at risk for delayed diagnosis [13].

Other studies have established that cholesterol, triglycerides, and lipoproteins may play a key role in prostate oncogenesis and severity. Moses et al. in 2009 reported that increased LDL serum levels were associated with a higher risk of prostate cancer in African American men but not in non-African American males [14]. However, Adedapo and collaborators in 2012, reported that lower serum levels of triglycerides and total cholesterol were associated with benign prostate hyperplasia and prostate cancer [15]. In that same year, Hayashi et al. found that high triglycerides serum levels were positively correlated to prostate cancer incidence in a sample of Japanese men age 60 years or older. In addition, they also reported that more aggressive prostate cancer cases defined as having a GS of 8 or greater were significantly associated with triglycerides levels above 150 mg/dL [16]. In 2009, Platz, E. et al.[17] followed 5,586 men in the placebo arm of the Prostate Cancer Prevention Trial (PCPT), and reported that in a group of men with GS 8–10, those with low serum cholesterol levels had a lower risk of high grade prostate cancer with worse prognosis. Work by Solomon et al. in 2008 [18] suggests an influence of cholesterol levels on the survival of cancer cells. Moreover the relationships between cholesterol levels and high grade prostate cancer seem to be dependent on BMI, with heavy men with low circulating cholesterol showing lesser rates of high grade and advanced disease [19]. Although the above mentioned research have brought light into the possible relationship of lipids and the aetiology and phenotype of prostate cancer, there has been limited participation of Hispanics in general and Puerto Ricans in particular as part of the studied cohorts.

Considering the high prevalence of metabolic syndrome in Puerto Rico, the BMI statistics, and the health disparity in prostate cancer mortality rates in this population, it is possible that lipid levels could influence the prostate cancer phenotype in this population. We found no evidence of a previous study associating serum lipid levels with the characteristics of prostate cancer in a Hispanic Puerto Rican population. Thus, in this exploratory study we tested the hypothesis that high triglycerides and low HDL levels were associated with a more severe cancer phenotype. This work contributes data that must be considered in the clinical management of PCa patients in a population of Hispanics, with a different ancestry background than other Hispanic and racial/ethnic groups [20].

## Results

Mean age for the cohort was 58.8 years (range: 40 to 75). Overall, 99 participants (49.8 %) were ≥ 60 years old, 157 (78.9 %) were overweight or obese, 73(36.7 %) had hypertriglyceridemia and 70 (35.2 %) had low HDL levels (Table 1). Low severity PCa [GS (3 + 4 or less) with a low pathologic stage (≤pT2c)] was found in 140 (70.4 %) patients, while 59 (29.6 %) had high severity PCa.

**Table 1:** Demographics and clinical characteristics of study sample (*n* = 199)

Characteristic	*n* (%)
Age at PCA diagnosis (years)	
<60	100 (50.25)
≥60	99 (49.75)
Mean age ± SD	58.82 ± 6.92
BMI (kg/m^2^)	
<25.00	42 (21.11)
25.00–29.99	98 (49.25)
≥30.00	59 (29.65)
Cholesterol (mg/dL)	
<200	125 (62.81)
≥200	74 (37.19)
Triglycerides (mg/dL)	
<150	126 (63.32)
≥150	73 (36.68)
HDL Cholesterol (mg/dL)	
≥40	129 (64.82)
<40	70 (35.18)
LDL Cholesterol (mg/dL)	
<160	177 (88.94)
≥160	22 (11.06)

Table 2 describes the demographic and clinical characteristics in patients by PCa severity. PCa severity varied significantly by age at cancer diagnosis, triglycerides, and HDL cholesterol levels (*p* < 0.10). In the low severity group, 97 (69.3 %) subjects had desirable HDL levels (≥40 mg/dL) compared with 43 (30.7 %) with low HDL levels (*p* = 0.042). Low triglycerides levels were present in 96 (68.46 %) of the subjects in the low severity group and in 29 (50.9 %) of the subjects in the high severity group (*p* = 0.018). We found a higher percentage (34 %) of high severity PCA in patients with high triglycerides and BMI < 30, and only in 20 % of patients with low triglycerides levels in the same BMI group. Table 3 shows the estimation of the unadjusted and adjusted OR (95 % CI) and PCa severity. In the unadjusted regression model, a higher BMI, age ≥ 60 years, high triglyceride levels and low HDL were significantly associated with PCa severity (*p* ≤ 0.10).

**Table 2:** Description of demographic and clinical characteristics by severity status of PCa

Characteristic	PCa Severity		*p*-value*
	Low (*n* = 140)	High (*n* = 59)	
	*n* (%)	*n* (%)	
Age at PCA diagnosis (years)			**0.007**
<60	79 (56.43)	21 (35.59)	
≥60	61 (43.57)	38 (64.41)	
BMI (Kg/m^2^)			0.216
<25.00	33 (23.57)	9 (15.25)	
25–29.99	70 (50.00)	28 (47.46)	
≥30.00	37 (26.43)	22 (37.29)	
Cholesterol (mg/dL)			0.508
<200	90 (64.29)	35 (59.32)	
≥200	50(35.71)	24 (40.68)	
Triglycerides (mg/dL)			**0.018**
<150	96 (68.57)	30 (50.85)	
≥150	44 (31.43)	29 (49.15)	
HDL Cholesterol (mg/dL)			**0.042**
≥40	97 (69.29)	32 (54.24)	
<40	43 (30.71)	27 (45.76)	
LDL Cholesterol (mg/dL)			0.465
<160	126 (90.00)	51 (86.44)	
≥160	14 (10.00)	8 (13.56)	

*p-value from Pearson Chi-square test. Figures in bold have statistical significance

**Table 3:** Logistic regression models of factors associated to the PCa severity among a sample of male patients in Puerto Rico

Characteristic	Unadjusted OR (95 % CI)	*p*-value	Adjusted OR (95 % CI)^a^	*p*-value
Age at PCa diagnosis (years)				
<60	1.0		1.0	
≥60	2.34 (1.25–4.40)	0.012	2.59 (1.34–5.02)	0.005
BMI (Kg/m^2^)				
<25.00	1.0		1.0	
25–29.99	1.47 (0.62–3.46)	0.381	1.26 (0.51–3.10)	0.614
≥30.00	2.18 (0.88–5.40)	0.092	2.28 (0.88–5.89)	0.089
Triglycerides (mg/dL)				
<150	1.0		1.0	
≥150	2.11 (1.13–3.93)	0.019	1.78 (0.91–3.49)	0.093
HDL Cholesterol (mg/dL)				
≥40	1.0		1.0	
<40	1.90 (1.02–3.56)	0.044	1.73 (0.87–3.42)	0.117

^a^Adjusted by all variables in the model. No interaction in the model, *p*-value from the Likelihood-ratio test = 0.4899

## Discussion

PCa is a complex disease with phenotypes that appear to arise as a result of “individualized” processes in each patient. Its development may be influenced by the personal lifestyle and nutritional habits of each individual [21]. Obesity and hyperlipidemia among other components of the metabolic syndrome are known risk factors for prostate cancer [22, 23]. The Puerto Rican population has unique characteristics in terms of nutritional habits and lifestyle. Ho and colleagues showed that the traditional diet of Puerto Ricans includes a low diversity of fruits and vegetables consumption and is, therefore, “unhealthful” [24]. Colon-Lopez, V. and collaborators in 2013 [25], found that in a group of 593 Puerto Ricans living in the island, only 19.5 % followed the recommendations for daily exercise activity and only 4.2 % met both the daily exercise and daily fruit and vegetables intake recommendations. Moreover 40.4 % of the respondents reported they did not believe exercise could lower their risk of cancer.

Abdominal obesity, hypertriglyceridemia, and low HDL levels have a high prevalence among the population living on the island [26]. Puerto Rican adult males are more likely to be overweight regardless of age as reported by Perez et al. [27]. Considering these phenotypes, and previous associations of lipid levels and PCa done by others in non-Hispanic populations, [28, 29] we aimed to establish if there was relationship between serum lipids levels and prostate cancer grade in Puerto Rican men. We found that high triglycerides and low HDL levels correlate each on its own with high severity prostate cancer. Associations lost significance upon multivariate adjustments.

## BMI and PCa severity

When we analyzed the association of presurgical BMI and PCa severity, our data suggest that in Puerto Rican overweight and obese men there was an increased risk for more severe disease. Nevertheless, the tendency is marginally significant. These results must be interpreted with caution considering the limitations imposed by using BMI as an overweight/obesity measurement. Despite its common use, BMI is not an exact measurement of body fat and does not consider body fat mass and its distribution. Therefore, it may misrepresent people with high lean body mass [30, 31]. In addition, a limitation of our study is that we do not have a longitudinal history of the patients’ BMI, which hinders our ability to establish a direct relationship between high BMI and the onset and development of prostate cancer severity.

## Triglycerides and PCa severity

When we analyzed the association of triglycerides and prostate cancer severity, our data suggest that in Puerto Rican prostate cancer patients with hypertriglyceridemia the risk of more severe cancer is increased. Triglycerides metabolism provides essential fatty acids [32]. It has been suggested that in prostate cancer the lack of regulation of LDL-receptor allows for the increased uptake of essential fatty acids when LDL serum levels are elevated. This provides a mechanism for Prostaglandin E2 (PGE2) synthesis, a known growth factor for cancer cells [32, 33]. The relationship between triglycerides and the severity could be explained by the contribution that high levels of triglycerides make to the essential fatty acids available for PGE2 production mechanism.

In addition, triglycerides levels include very low density lipoproteins (VLDL), chylomicrons and remnant lipoproteins (RLP), produced after the hydrolysis of chylomicrons and VLDL [34]. These RLPs bind to the cells via the Apo E and ApoB/E receptors. In vitro studies have shown that 1 μg/mL of RLP induces the proliferation of PC-3 prostate cancer cells, in a dose dependent manner. This dose is comparable to levels of 10 ng/dL that have been detected in patients [35]. These results should be further characterized in an *in vivo* model.

## Total Cholesterol, HDL and PCa severity

It is known that cholesterol plays an important role in prostate cancer as a precursor of androgens, cell proliferation mediator and inflammation [36]. Cholesterol is also included within the lipid bimolecular layer of the cell membrane, and this includes prostate cancer cells [37]. We did not find statistically significant differences in serum total cholesterol levels among the high and low severity prostate cancer. These results diverge with previous studies [17, 28] which finds positive correlations between lower cholesterol levels and lower risk of high-grade cancer or studies associating cholesterol with tumor growth in animal models [38–40]. It is important to notice that among the many studies done to look for associations between cholesterol levels and PCa or the use of cholesterol reducing drugs, ie. statins and PCa there are no standardized measurements for PCa grade or severity. Some studies use only the GS, in two categories higher or lower than 7 [32] others use three [28] and still others [17] as we do here, include the tumor stage as part of the considerations to assess the severity of the disease. Thus, the results are actually not fully comparable among the studies.

The formation of lipid rafts in PCa cells have been postulated to facilitate signaling in cancer cells that foster carcinogenic transformation throughout time. Admittedly, our cholesterol findings are limited by the lack of longitudinal data regarding the patients’ cholesterol levels, thus we cannot assess the effect of the range of time that the patient had high or low cholesterol levels and the PCa phenotype. In addition, the role of other cholesterol susceptible pathways in carcinogenesis must be addressed.

In our study group, a higher percentage of patients with high HDL levels had low severity PCa. It has been suggested that HDL plays a protective role in the pathophysiology and cancer progression [41]. Low HDL effect could be explained by reduced binding of PON-1(paraoxonase) thus reducing PON-1free radical scavenging capacity [42]. Additionally, HDL may convey some protection from cancer severity by inhibiting the formation of lipid rafts which have been associated to procarcinogenic cell signaling through the activity of Caveolin-1(Cav-1) [43].

## Conclusions

Undeniably, our study suffers from the limitations associated to a small study group. It is not a longitudinal and prospective study, thus the study design could not include an assessment of lifestyle habits that may act as confounders.

Notwithstanding the limitations imposed by sample size and lack of longitudinal data, this study sheds light into the possible effects of lipids in the PCa phenotype of an understudied population, with high overweight and PCa mortality rates. Our data was not gathered island wide but the treatment facility where we conducted our study receives patients from all over the island, thus the population, although small is not confined to the metropolitan area of San Juan. Moreover, more than 95 % of the patients had some type of medical insurance, therefore we are confident that the possible effect of lack of access to medical care was probably not present in the studied group. The potential clinical implication is that, triglycerides and HDL levels may be related to PCa severity and therefore relevant to patient management, remarking that assessment of these lipid levels should be part of the routine screening and diagnostic procedures. We would like to conclude with the following: There is a need for prospective, longitudinal and well-designed studies, with adequate representation of ethnic and racial diversity to elucidate the role of HDL and LDL and total cholesterol levels, in the risk of developing PCa and in its progression.

## Methods

### Ethics

This study was approved by the Institutional Review Board (IRB) of the University of Puerto Rico Medical Sciences Campus (IRB approved protocol #8860211).

### Study population

The studied population was chosen from patients undergoing treatment at a private medical facility in San Juan, Puerto Rico which receives patients from all-over the island and performed 516 radical prostatectomies (RP) during the study period 2005–2012.

Inclusion criteria for the study were: Patients had to be Puerto Rican, defined as being born in Puerto Rico with parents and grandparents also from Puerto Rico, aged 40–75 years old. Importantly, Radical prostatectomy (RP) was the first treatment for the PCa. Patients were excluded if they had been diagnosed previously with other cancers or had viral infections at the time of the first screening visit. Four hundred and eighty one (481) patients’ records complied with these requirements. Out of the 481, we excluded 267 records that did not have the lipid panel data. We also excluded patients records (*n* = 15) that were under statin treatment for cholesterol management. Ultimately 199 records were included in the study.

### Data collection procedures

The following clinical information was recorded from medical charts: age, fasting serum lipid levels at the time of their diagnosis, pre-radical prostatectomy PSA, height, weight, and prostatectomy GS and Pathologic Stage from pathology report. Lipid data accrued included triglycerides, cholesterol, and lipoproteins (HDL and LDL). Lipid levels were recorded from the patients’ first visit to the facility prior to any intervention. Lipids were categorized following the US National Institutes of Health - National Cholesterol Education Program ATP III guidelines (Table 4) [44]. Height and weight were used to calculate BMI according to Centers for Disease Control formula (weight [kg]/height^2^ [m^2^]). BMI categories were defined as underweight/normal (≤24.9 kg/m^2^), overweight (25.0–29.9 kg/m^2^), and obese (≥30.0 kg/m^2^).

**Table 4:** Lipid levels categories following the US National Institutes of Health - National Cholesterol Education Program ATP III guidelines

Triglycerides	Desirable: <150 mg/dL	Non desirable: >150 mg/dL
Cholesterol	Desirable: <200 mg/dL	Non desirable: >200 mg/dL
HDL	Low: <40 mg/dL	High: >40 mg/dL
LDL	Low: <160 mg/dL	High: >160 mg/dL

Disease severity was defined using both the prostatectomy GS and the American Joint Committee on Cancer (AJCC) prostate cancer Pathologic Staging [45]. Low severity was defined as GS ≤7 (3 + 4) and pathologic stage ≤ pT2c. High severity was defined as GS ≤7 (3 + 4) and pathologic stage ≥ pT3a or GS ≥7 (4 + 3) and pathologic stage > pT2c.

## Statistical analysis

Univariate analysis was performed to characterize the population (*n* = 199) according to demographic and clinical characteristics. Contingency tables were generated to assess the relationship of demographic and clinical characteristics with prostate cancer severity using Pearson chi-square test. Unadjusted and adjusted logistic regression models were fitted to estimate the odds ratios (ORs) with 95 % confidence intervals (CI) to define these relationships. Variables statistically associated with PCa severity (*p* < 0.10) in the unadjusted logistic regression models were included in the multivariate logistic regression model. Considering the sample size we used a level of *p* ≤ 0.10 as a screening criterion for variable selection for the multivariate logistic regression model, to which a level of *p* ≤ 0.05was assigned. This statistical and epidemiological decision was based on recommendations done by Bendel and Afifi [46] and Mickey and Greenland [47] on logistic regression. These authors show that use of a more traditional level (such as 0.05) often falls to identify variables that have clinical and scientific significance. All analyses were performed using STATA 12.0 (College Station, Texas, USA)

## Abbreviations

PCa: Prostate cancer
BMI: Body mass index
PSA: Prostate-specific 1ntigen
GS: Gleason score
BRFSS: Behavioral risk factor surveillance system
IRB: Institutional Review Board
HIV: Human immunodeficiency virus
HCV: Hepatitis C virus
HDL: High density lipoproteins
LDL: Low density lipoproteins
AJCC: American Joint Committee on Cancer
OR: Odds ratios
CI: Confidence intervals
PGE2: Prostaglandin E2
VLDL: Very low density Lipoproteins
RLP: Remnant lipoproteins
PON-1: Paraoxonase
Cav-1: Caveolin-1
NIH: National Institutes of Health
US: United States of America
